# A salient region detection model combining background distribution measure for indoor robots

**DOI:** 10.1371/journal.pone.0180519

**Published:** 2017-07-24

**Authors:** Na Li, Hui Xu, Zhenhua Wang, Lining Sun, Guodong Chen

**Affiliations:** Robotics and Microsystem Center, Soochow University, Suzhou, Jiangsu, China; National University of Defense Technology College of Mechatronic Engineering and Automation, CHINA

## Abstract

Vision system plays an important role in the field of indoor robot. Saliency detection methods, capturing regions that are perceived as important, are used to improve the performance of visual perception system. Most of state-of-the-art methods for saliency detection, performing outstandingly in natural images, cannot work in complicated indoor environment. Therefore, we propose a new method comprised of graph-based RGB-D segmentation, primary saliency measure, background distribution measure, and combination. Besides, region roundness is proposed to describe the compactness of a region to measure background distribution more robustly. To validate the proposed approach, eleven influential methods are compared on the DSD and ECSSD dataset. Moreover, we build a mobile robot platform for application in an actual environment, and design three different kinds of experimental constructions that are different viewpoints, illumination variations and partial occlusions. Experimental results demonstrate that our model outperforms existing methods and is useful for indoor mobile robots.

## Introduction

Due to population ageing [1], the cost of health care is raising in recent years and indoor robots will play significant roles in our daily life. Vision system, the most important perception vehicle for indoor robot, obtains a flood of visual information to perceive and understand the surrounding world, but as is often the case that only a few regions are relevant to a given context. Therefore, indoor robot is expected to possess the information management skill to capture useful visual information regard to a specific task. Inspired by the primate-customized visual attention mechanism, which is simple, yet extremely robust: select the most relevant information among the plethora of visual information [2, 3], saliency detection has been extensively studied to deal with the enormous amount of information and enhance computational efficiency. It has been used in many applications including object recognition [4–6], image segmentation [7, 8], image retrieval [9], visual tracking [10–12] and human-robot interaction (HRI) [1, 13–19].

**Fig 1:**
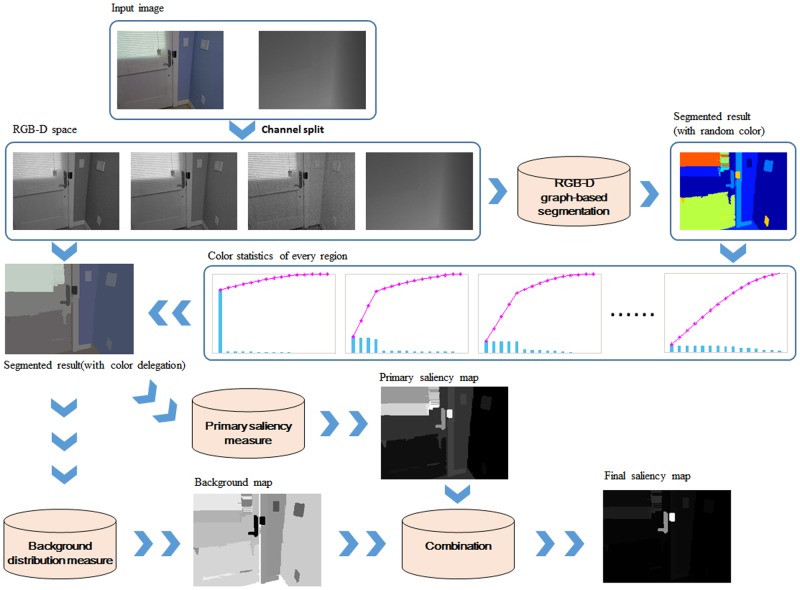
The framework of the proposed method. Firstly, to keep the completeness and compactness of salient region candidates, an input image is divided into regions using color(RGB) and depth cues. Secondly, a color delegation are adaptively selected for each region and used to measure the primary saliency. Thirdly, the background distribution is assigned by the spatial distribution and the compactness of each region. Finally, the primary saliency map and the background distribution map are combined to reduce the false positive of background regions and false negative of salient regions.

Plenty of saliency detection methods proposed have tested on the public datasets and performed well. However, to be the best of our knowledge, most of the existing methods hold true for natural images with simple background and single salient object, but they cannot work well for indoor scene with complex backgrounds, several salient objects and illumination variations. We also notice that most of the proposed methods are based on visual features in 2D scenes, and the depth information is ignored even though it is essential cues for human beings to perceive the world. Inspired by [20–22], we propose a new combined salient region detection model that integrates background distribution into primary saliency. The framework of the proposed method can be seen in Fig 1.

The rest of this paper is organized as follows. Related works are provided in Section 2. Our model is descried in detail in Section 3. Experiments on two public datasets and the robot platform are introduced and analyzed in Section 4 and Section 5, respectively. Conclusions and future work are listed in Section 6.

## Related work

In this section, related work of saliency object detection are introduced in two aspects: computer vision and robot application.

Currently, salient region detection methods proposed in the field of computer vision have sprung up and gained widespread popularity in many applications including robots. They utilize some different features (such as color, intensity, edge, face and person) to determine the possibility to be attended of a pixel [20, 21, 23–25], superpixel [26, 27] or region [20, 22, 28, 29]. The spatial attention model by Zhai [23] computes a pixel-level saliency map by the intensity histogram of input image, whose attended regions are detected by the region growth technique and marked with bounding boxes. Following in Zhai [23], two models proposed by Cheng [20], the histogram-based contrast (HC) and the region-based contrast(RC), perform well in natural images. In HC, saliency is measured for each pixel by color contrast to all other pixels. In RC, after graph-based segmentation [30] a region’s saliency is computed by color contrast and spatial distance to others in the image. The frequency-tuned (FT) saliency detection approach [25] uses band-pass filtering to produce full-resolution saliency maps. Its low computational complexity makes it highly suitable for real-time applications on a mobile robot. In Federico’s model [26], the input image is abstracted into elements using the SLIC superpixel technique and the saliency estimation is obtained from the uniqueness and the spatial distribution of these elements. Developed from Itti model, the model proposed by Wang [29] assigns saliency based local and global saliency information of each segmented region using color and orientation feature. An optimization framework presented by Zhu [22] combines background detection, and boundary connectivity is proposed to quantify how heavily a region is connected to image boundaries to ensure high accuracy background detection. An method by Jiang [21] is proposed to detect salient regions by mapping pixels into foreground and background regions in RGB-D images.

Visual saliency models for robots could be generally divided into two categories: overt visual attention models and covert visual attention models. For the overt visual attention model, the research works concentrate on the camera maneuvering mechanism based on the principle of overt visual attention [31]. In [14], a fast approximation to a Bayesian model of visual saliency is proposed to orient a camera as quickly as possible toward human faces. Results show that it can provide saliency maps in about 10ms per 160 ×120 pixel video frame. For the real-time implementation, they only use image intensity channel, not color channels As described in detailed previously [32], a saliency-based “lazy” approach is proposed for scene exploration of newly entered rooms that reduces the amount of necessary head movement while it strongly favors to attend the most salient proto-objects as soon as possible. For the covert attention model, they give emphasis on a sensory stimulus mentally without changing gaze direction. In the intelligent system described in [19], vision saliency is used to restrict the total number of possible gazes to a smaller set that still contains salient objects. In [17] a biologically inspired vision system is presented for human-robot interaction. In order to reduce clutter and improve gesture recognition rates, visual saliency, computed by color, motion and disparity cues, is used to segregate hands from the background. A real-time parallel model for saliency maps in [18] is put forward to predict gaze directions. IMAPCAR2, an image recognition processor with advanced parallel processing capabilities, is exploited to improve the operating efficiency. In [21] an effective method is proposed to detect salient regions by mapping pixels into foreground and background regions in RGB-D images.

## Methods

**Fig 2:**
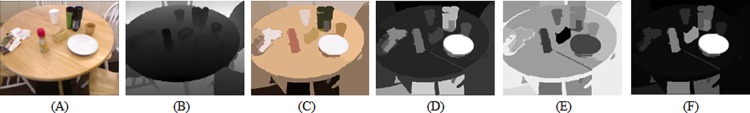
An illustrative example of our method. A: RGB image. B: depth map. C: the segmented result. For display, each region is shown in the mean color. Note that in our model, each region is assigned with a color delegation that is obtained by picking more frequently occurring colors. D: the primary saliency map. E: the background distribution map. F: the final saliency map.

In this section, we propose a new approach for salient region detection with the combination of primary saliency and background distribution. The illustration of the proposed method is shown in Fig 2. In the following subsections, each step will be presented in detail.

### Segmentation

It is essential for indoor robots to perceive the surrounding environment, localize desired objects, and know their shapes and sizes. Therefore, keeping the completeness of salient objects is the premise and guarantee to accomplish tasks for indoor robots. Obviously, Pre-segmentation is an effective approach to meet the requirement. Depth information is demonstrated to well hold the integrality of foreground objects and strong contours in the input image even in an intricate scenario. Therefore, the graph-based RGB-D segmentation [21, 30] is introduced taking advantage of depth and color information. Thanks to the segmentation, salient region candidates tend to be complete.

The segmentation example is shown in Fig 2C. Notice that the segmented result in mean color is just for display. It can be seen that each object on the table is segmented into one single region nearly. For example, the glass bottle with a red lid and a white label is separated into one region. It implies that graph-based RGB-D segmentation can capture completeness of objects excellently. Completeness of objects and homogeneous color property of regions is inherently a pair of contradiction and it is difficult to balance them, therefore we discard general representation which uses mean color values to characterize each region, and adopt a new depiction that some representative colors of each region are picked adaptively. Details of the representative colors selectivity and primary saliency measure are described in the next subsection.

### Primary saliency measure

The commonly employed assumption, regions which stand out from other regions in the image tend to catch human attention and are regarded as salient, supports the implementation of recent contrast based saliency measure [20–24, 26, 29]. Saliency maps of these approaches are calculated based on some visual features, such as color, depth, orientation and contour, or spatial distribution. Here a primary saliency map and a background distribution map are calculated using two kinds of visual features (color and depth) and spatial layout of each region, respectively.

**Fig 3:**
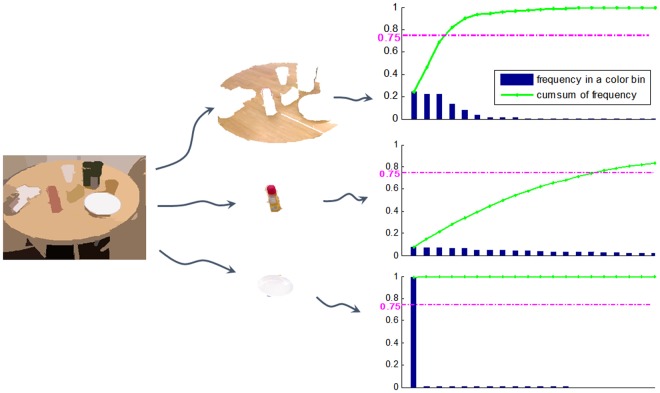
An example of the color delegation. The segmented result (first column) presented by mean color values, three segmented regions (second column) by original RGB information and color statistics (third column) of respective regions where the blue histogram is sorted in the non-increasing order of color occurrence frequency, and the green line with asterisk mark represents the cumulative sum of the corresponding histogram. The color delegation of a region consists of more frequently occurring colors which cover more than a certain proportion of this region area. We introduce a threshold for the color delegation selection, and it is set to 0.75 as shown by the magenta dashed line. For each region, intuitively, color bins below the magenta dashed line and the next one compose its color delegation.

In order to keep the comprehensiveness of color information and balance it with the computational efficiency, we build a color histogram for each region and pick more frequently occurring colors as the color delegation. Specifically, each color channel in RGB color space is first quantized to *N* bins [20] so that the total color number drops from 256^3^ to *N*^3^, and a *N*^3^-bin histogram is built to denote color properties for each region. After these bins are sorted in the non-increasing order, more frequently occurring colors which cover more than *φ* of this region area will be selected as its color delegation. An example result is shown in Fig 3 where we set *N* = 8 and *φ* = 0.75. The example shows that the glass bottle can be described by 15 color values, while the white plate can be depicted by only one.

Thanks to the region representation based on the color delegation, the primary saliency can be measured next. Salient regions should be distinctive and high-contrast compared with other regions in the image. We evaluate the primary saliency by contrasting colors of a region with all other regions,

$$\begin{array}{c} RS\left(r_{i}\right)=\sum_{i\neq j}\omega_{j}*D_{c}\left(i,j\right), \end{array}$$ (1)

in which *D*_*c*_(*i*, *j*) is the color distance of region *r*_*i*_ and *r*_*j*_ with the color delegation $c_{i}=\{c_{i}^{1},c_{i}^{2},\ldots \ldots ,c_{i}^{n_{i}}\}$ and $c_{j}=\{c_{j}^{1},c_{j}^{2},\ldots \ldots ,c_{j}^{n_{j}}\}$ respectively. Region *r*_*i*_(*r*_*j*_) has *n*_*i*_(*n*_*j*_) representative colors and each representative color value is obtained by the mean value of pixels dropping into the relevant bin.

$$\begin{array}{c} D_{c}\left(i,j\right)=\sum_{p=1}^{n_{i}}\sum_{q=1}^{n_{j}}f\left(c_{i}^{p}\right)*f\left(c_{j}^{q}\right)*∥c_{i}^{p}-c_{j}^{q}∥, \end{array}$$ (2)

in which $c_{i}^{p}$ is the p-th representative color of region *r*_*i*_ and $f(c_{i}^{p})$ is the occurrence frequency of $c_{i}^{p}$ in region *r*_*i*_. Here we utilize occurrence frequencies as the weight of color distance between two representative colors to emphasize high-frequency ones.

$$\begin{array}{c} \omega_{j}=AR\left(r_{j}\right)*e^{-\alpha *D_{s}\left(r_{i},r_{j}\right)}. \end{array}$$ (3)

In Eq (1), *ω*_*j*_ is the weight of region *r*_*i*_ and influenced by two factors *AR*(*r*_*j*_) and *D*_*s*_(*r*_*i*_, *r*_*j*_). *AR*(*r*_*j*_) is the area ratio of region *r*_*j*_ in the input image and *D*_*s*_(*r*_*i*_, *r*_*j*_) is the distance between centroids of region *r*_*i*_ and *r*_*j*_. *α* is the scaling factor to control the strength of *D*_*s*_(*r*_*i*_, *r*_*j*_).

### Background distribution measure

In indoor environments with complex backgrounds, several salient objects and illumination variations, the global contrast method based on saliency measure often causes some undesired outcomes. For instance, in Fig 2D the floor intuitively regarded as region of background is labeled with relatively high saliency values, while the carton close to the table in color domain isn’t underscored.

**Fig 4:**
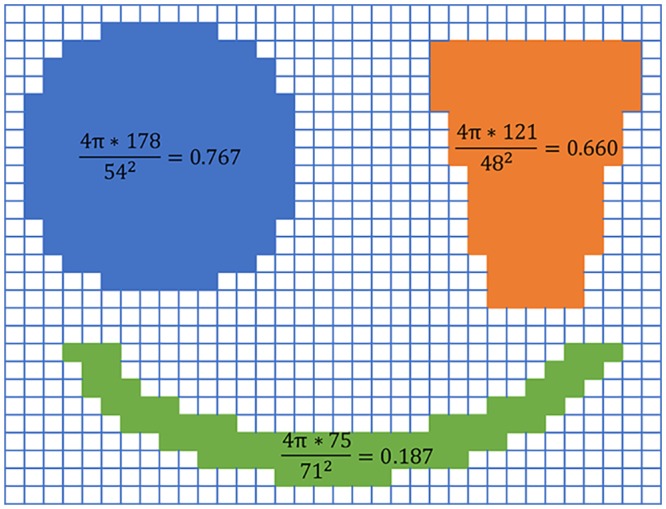
An illustration of region roundness. The synthetic image comprises three regions with different compactness. Each region roundness value is displayed on it.

We discover that, generally, salient object regions have the property of compact sizes while background ones distribute widely and near image boundaries. Based on the hypothesis, background distribution *BD*(*r*_*i*_) is introduced to reduce and even exclude false negative and false positive from the previous step,

$$\begin{array}{c} BD\left(r_{i}\right)=\omega_{BD}\left(r_{i}\right)*e^{-\beta *rd(r_{i})}, \end{array}$$ (4)

in which *ω*_*BD*_(*r*_*i*_) is the Gaussian weight of region *r*_*i*_, and *β* controls the strength of the region roundness. *rd*(*r*_*i*_), called region roundness, is a new proposed measure to describe the compactness of a region. It is defined as:

$$\begin{array}{c} rd\left(r_{i}\right)=\frac{4\pi *A(r_{i})}{L_{c}{\left(r_{i}\right)}^{2}}, \end{array}$$ (5)

in which *A*(*r*_*i*_) and *L*_*c*_(*r*_*i*_) are area and contour length of region *r*_*i*_ respectively. The more this value is, the more compact relevant region is. Region roundness is usually large for salient object regions and small for background regions. Fig 4 can support our inference where the blue region which we believe the most compact has the maximum of region roundness and the green one which is dispersed apparently is allocated a smallest value. Therefore, the region roundness measure is available to quantify region characteristics.

Spatial layout in images have the universality that background regions can be easily connected to image boundaries while foreground objects cannot. Utilizing boundary connectivity [22] and distance to the image center of each region, we set a valid weight *ω*_*BD*_(*r*_*i*_) to influence background distribution measure.

$$\begin{array}{c} \omega_{BD}\left(r_{i}\right)=1-e^{-\{\frac{BC(r_{i})}{\delta_{BC}}+\frac{DC(r_{i})}{\delta_{DC}}\}}, \end{array}$$ (6)

in which *DC*(*r*_*i*_) is the distance between the image center and region *r*_*i*_, and *BC*(*r*_*i*_) is the boundary connectivity of region *r*_*i*_ to quantify how heavily a region is connected to image boundaries. *δ*_*BC*_ and *δ*_*DC*_ control the strength of weighting of *BC*(*r*_*i*_) and *DC*(*r*_*i*_) respectively. Boundary connectivity is first defined in Zhu’s model [22] as:

$$\begin{array}{c} BC\left(r_{i}\right)=\frac{L_{b}\left(r_{i}\right)}{\sqrt{A(r_{i})}}. \end{array}$$ (7)

For simplicity and efficiency, *L*_*b*_(*r*_*i*_) is set to be the pixel numbers along image boundaries in region *r*_*i*_.

### Combination

By now, we have already obtained a primary saliency map and a background distribution map. To inhibit false positive of background regions and raise false negative of salient regions, we combine the two maps in a way of an exponential function:

$$\begin{array}{c} Sal\left(r_{i}\right)=RS\left(r_{i}\right)*e^{-\gamma *BD(r_{i})}. \end{array}$$ (8)

Note that primary saliency *RS*(*r*_*i*_) and background distribution *BD*(*r*_*i*_) are both normalized. The parameter *γ* is the scaling factor for the exponential to control the range of the background distribution measure.

As can be seen in Fig 2, input images ((A) and (B)) are first merged into homogeneous regions as in (C). Two samples of primary saliency map and background distribution map are shown in (D) and (E). The resulting saliency map is shown in (F).

## Experiments on datasets

### Datasets

The proposed model is aimed at salient object detection in the complicated indoor environment. To evaluate the proposed model, we select two datasets with complex scenes: DSD [33] and ECSSD [28].

DSD dataset [33] comprises 80 color and depth image pairs with associated pixel-wise ground truth segmentation masks. The dataset is obtained in a real-world indoor environment and used for studies of depth-based salient detection (such as [21]). Scenes in this dataset are assigned to multiple foreground objects of potential interest and complex background structure.

ECSSD [28] dataset consists of 1000 color images with associated pixel-wise binary masks. It includes a large number of semantically meaningful but structurally complex natural images [34]. Note that ECSSD don’t cover any depth images, the reason we select this dataset is just to check the performance and universality of our model in some complex scenes. Depth information is ignored in the experiments with ECSSD, namely the graph-based RGB-D segmentation turns into the ordinary graph-based RGB segmentation.

### Evaluation criteria

The precision-recall curve (PR curve), F-measure and the mean absolute error (MAE) [34], three universally-agreed, standard and easy-to-understand measures, are selected to evaluate the detection accuracy of the proposed model.

The PR curve is based on the overlapping area between saliency prediction and ground truth segmentation masks. For a saliency map, a fixed threshold which changes from 0 to 255 is set to convert *S* to a binary mask *M*, and *Precision* and *Recall* are computed by comparing *M* and ground truth *G*,

$$Precision\;=\;\frac{∥M\cap G∥}{∥M∥},$$ (9)

$$Recall=\frac{∥M\cap G∥}{∥G∥},$$ (10)

where ∥•∥ represents the number of non-zero entries in the mask. On each threshold, a pair of precision/recall scores are computed, and are finally combined to form a PR curve. The PR curve is commonly used to reliably compare how well various saliency maps highlight salient regions in images.

Besides, proposed by Achanta [25], the another way to partition a saliency map *S* is to use an image-dependent adaptive threshold, which is computed as twice as the mean saliency of *S*,

$$\begin{array}{c} T_{a}=\frac{2}{W*H}\sum_{x=1}^{W}\sum_{y=1}^{H}∥S(x,y)∥. \end{array}$$ (11)

where *W* and *H* are the width and the height of the respective saliency map. And this mode of calculating *Precision* and *Recall* will be used by *F*_*measure*_.

Usually, neither *Precision* nor *Recall* can comprehensively evaluate the quality of a saliency map. To this end, the *F-measure* is proposed as a weighted harmonic mean of them with a non-negative weight *ζ* [35]:

$$\begin{array}{c} F_{measure}=\frac{(1+\zeta^{2})*Precision*Recall}{\zeta^{2}*Precision+Recall}. \end{array}$$ (12)

As suggested by many salient region detection works [20, 25, 26], *ζ*^2^ is set to 0.3 to increase the importance of the *Precision* value. The reason for weighting precision more than recall is that recall rate is not as important as precision [24]. Here, the saliency map *S* can be binarized with the threshold from the Eq (11), and the presion and recall values can be calculated by the Eq (9) and the Eq (10).

The overlap-based evaluation measures introduced above do not consider the true negative assignments of the saliency map, i.e., pixels correctly marked as non-salient. For a more comprehensive comparison, the MAE is introduced as the second evaluation criteria to compensate the PR curve, and is used in recent methods [21, 22, 26, 34]. The *MAE* aims to measure how close a saliency map *S* is to the ground truth *G*,

$$\begin{array}{c} MAE=\frac{1}{W*H}\sum_{x=1}^{W}\sum_{y=1}^{H}∥S(x,y)-G(x,y)∥, \end{array}$$ (13)

where *S* and *G* are both normalized to the range [0, 1].

### Parameter evaluation

The proposed model brings in several parameters that are *α* in the Eq (3), *β* in the Eq (4), *γ* in the Eq (8) and *δ*_*BC*_ and *δ*_*DC*_ in the Eq (6). To investigate the effect of these parameters in the model [36], we conduct some experiments using DSD dataset.

**Fig 5:**
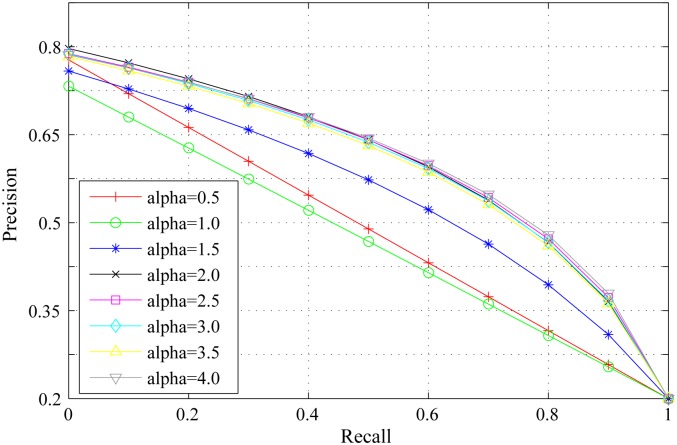
PR curves for different *α* on the DSD dataset.

**Fig 6:**
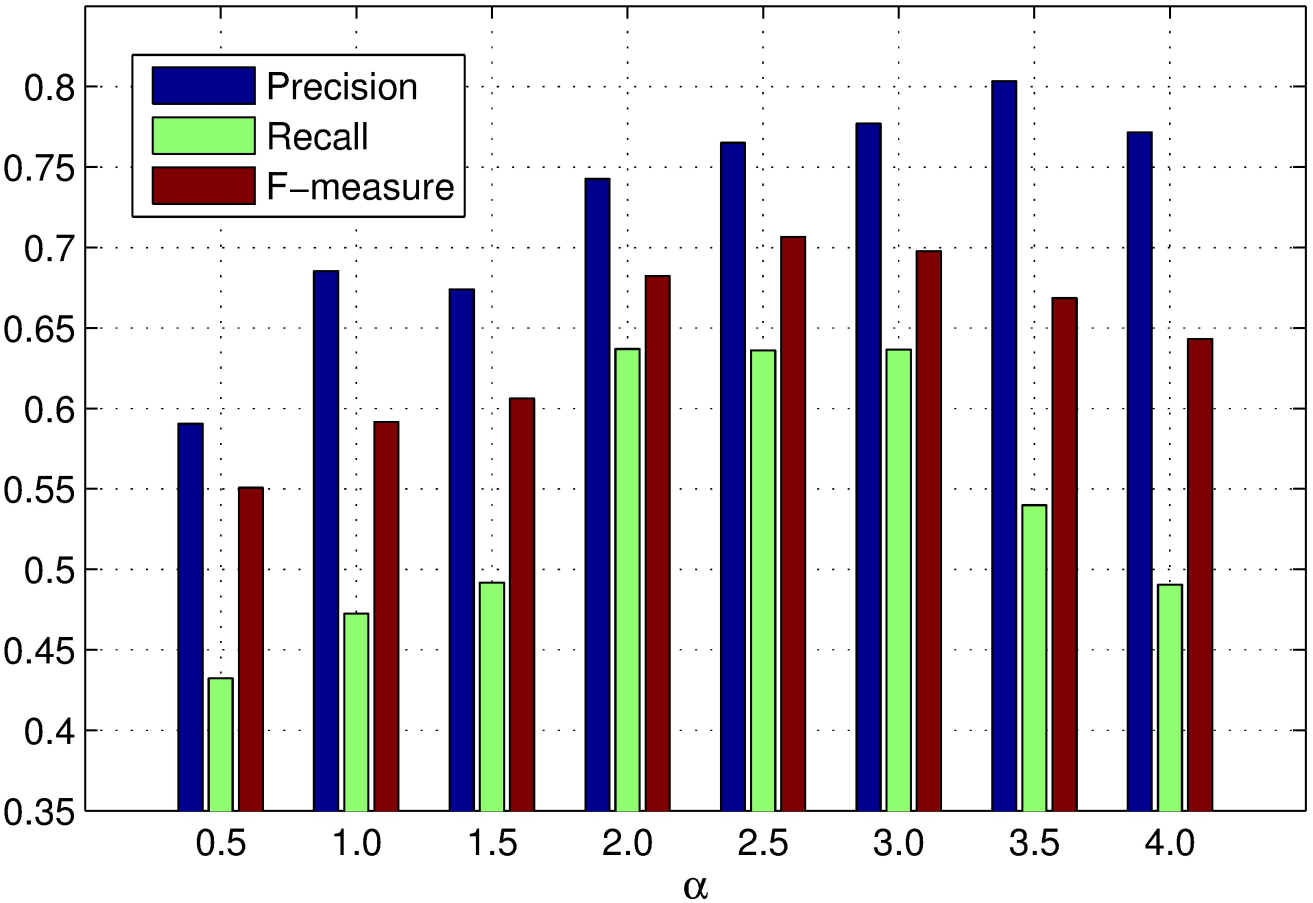
F-measure for different *α* on the DSD dataset.

**Fig 7:**
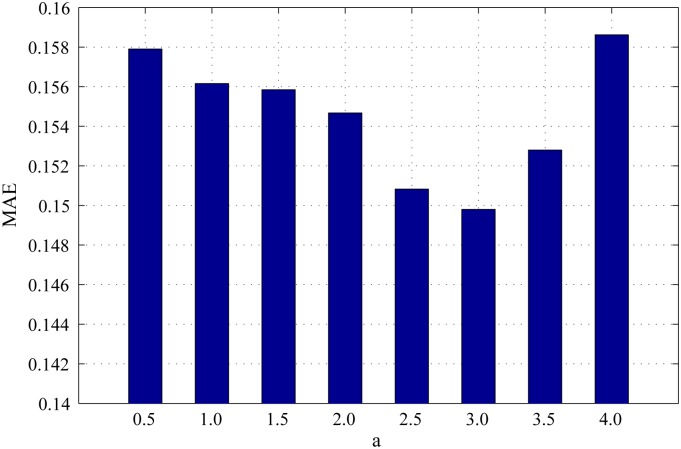
MAE for different *α* on the DSD dataset.

*α* is the scaling factor to control the strength of spatial distance between regions. If *α* is too small, the weight of spatial distance between regions($e^{-\alpha *D_{s}(r_{i},r_{j})}$ in the Eq (3)) will increase(close to 1), which will lead to expand the influence of spatial features during the primary saliency measure. If *α* is too large, the influence of spatial features will reduce during the primary saliency measure. Figs 5, 6 and 7 show the PR curves, F-measure and MAE for different *α* on the DSD dataset respectively. When *α* is greater than 2.0, the performance of different PR curves is similar. According to bar charts of F-measure and MAE, the performance reaches its peak when *α* = 3.0. The performance improves gradually with *α* when *α* is smaller than 3.0 and reduces speedily with *α* when *α* is larger than 3.0. Therefore, we set *α* = 3.0 through the experiment process.

**Fig 8:**
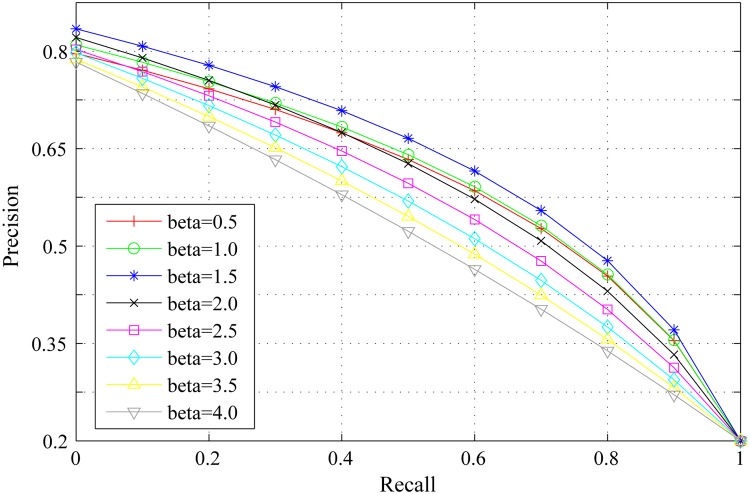
PR curves for different *β* on the DSD dataset.

**Fig 9:**
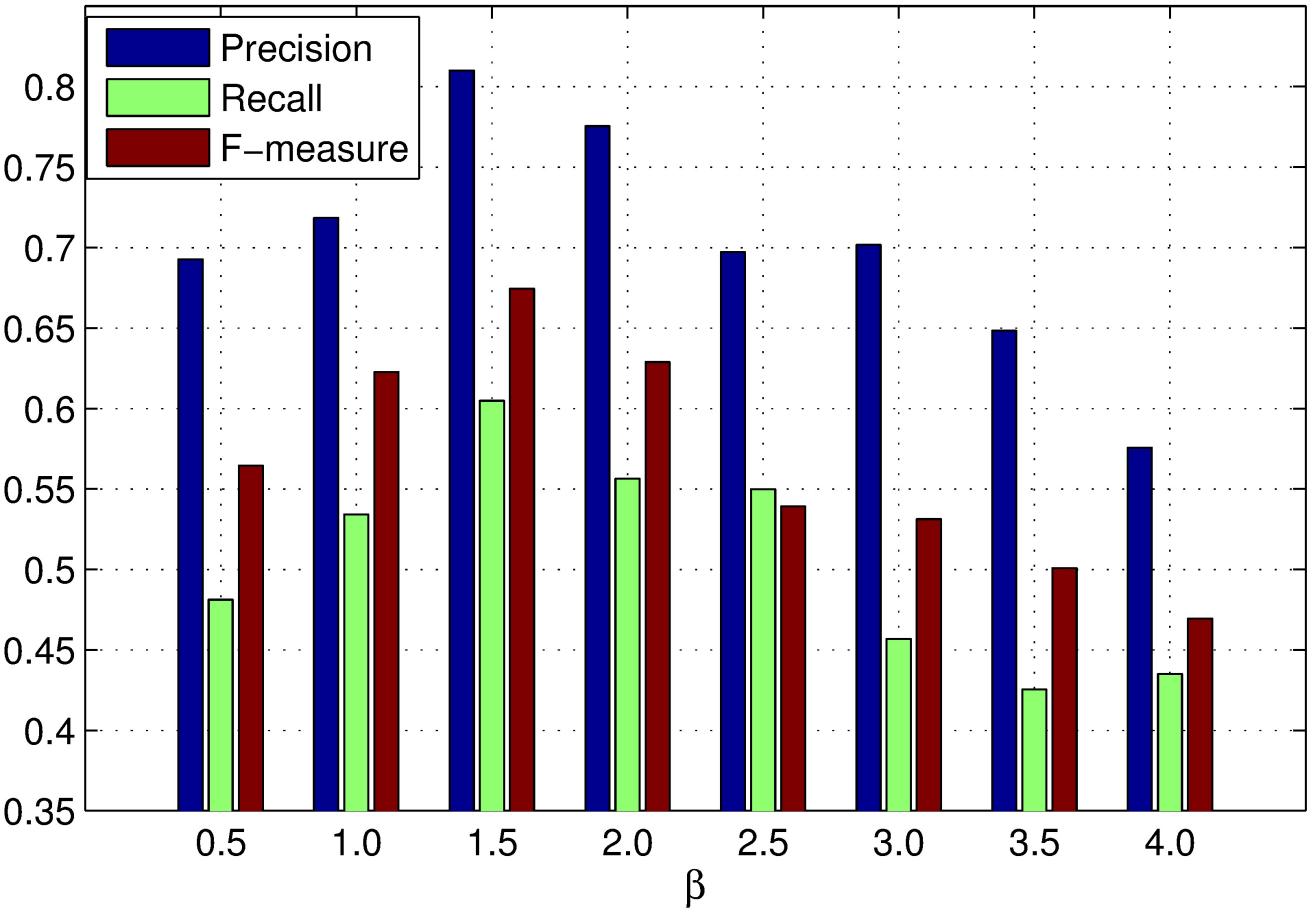
F-measure for different *β* on the DSD dataset.

**Fig 10:**
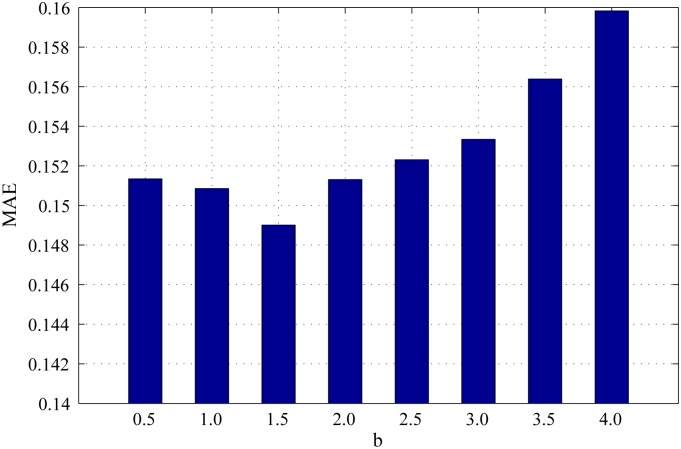
MAE for different *β* on the DSD dataset.

*β* is the scaling factor to control the strength of region roundness. The influence of *β* on region roundness is the same as that of *α* on spatial distance between regions. Figs 8, 9 and 10 show the PR curves, F-measure and MAE for different values of *β* on the DSD dataset respectively. In terms of PR curves, F-measure and MAE simultaneously, the performance reaches its peak as *β* = 1.5. When *β* is smaller than 1.5, the performance improves steadily with *β*. When *β* is larger than 1.5, the performance reduces speedily as *β*. Therefore, we set *β* = 1.5 in all experiments.

**Fig 11:**
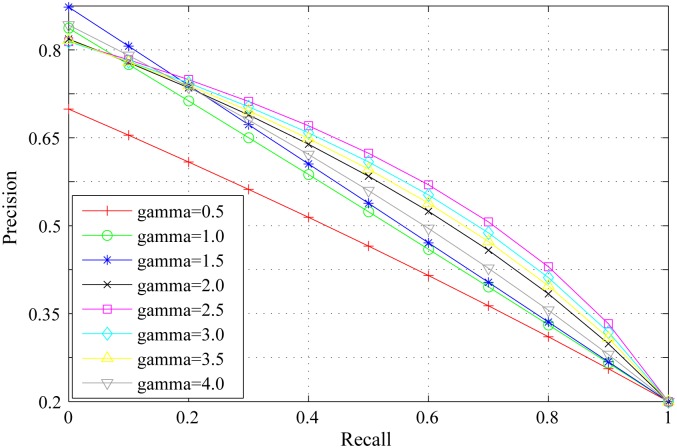
PR curves for different *γ* on the DSD dataset.

**Fig 12:**
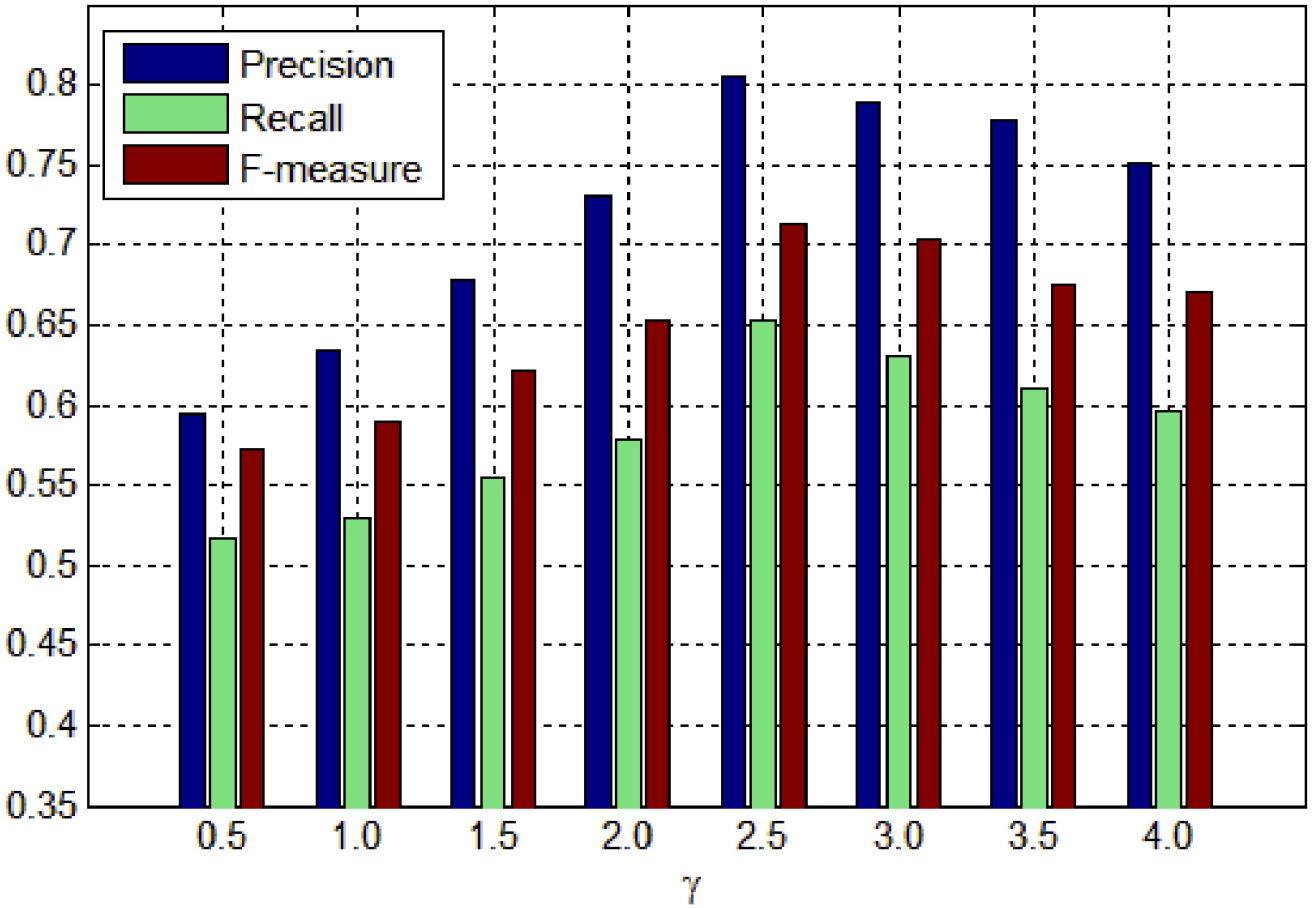
F-measure for different *γ* on the DSD dataset.

**Fig 13:**
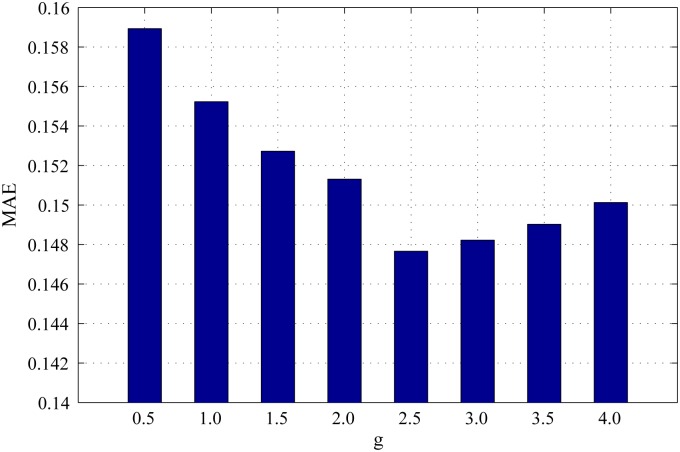
MAE for different *γ* on the DSD dataset.

*γ* is the scaling factor to control the range of background distribution measure. The influence of *γ* on background distribution measure is the same as that of *α* on spatial distance between regions. Figs 11, 12 and 13 show the PR curves, F-measure and MAE for different values of *γ* on the DSD dataset respectively. In terms of PR curves, F-measure and MAE simultaneously, the performance reaches its peak when *γ* = 2.5. When *γ* is smaller than 2.5, the performance increases steadily with *γ*. When *γ* is larger than 2.5, the performance reduces gradually with *γ*. Therefore, we set *γ* = 2.5 in all experiments.

In addition, *δ*_*BC*_ and *δ*_*DC*_ control the strength of boundary connectivity *BC*(•) and distance between the image center and a region *DC*(•). This two parameters jointly constitute a Gaussian weight *ω*_*BD*_(•) to impact background distribution measure. To obtain reasonable value of *ω*_*BD*_(•), *δ*_*BC*_ and *δ*_*DC*_ are adaptively set as the maximum of *BC*(•) and *DC*(•) for every image. Let us consider two extreme situations: When a region is far away from image center and connected to image boundaries, *BC*(•) and *DC*(•) of the region both approach the corresponding maximums, which will lead to approximately the largest Gaussian weight of background distribution measure in the region(*ω*_*BD*_ ≈ 1 − *e*^−(1+1)^ ≈ 0.865). Consequently, background distribution *BD*(•) of the region tends to obtain a bigger value, which means that the region may belong to background areas. When a region is close to image center and separated from image boundaries, *BC*(•) and *DC*(•) of the region are both close to 0, which will lead to approximately the smallest Gaussian weight of background distribution measure in the region(*ω*_*BD*_ ≈ 1 − *e*^−(0+0)^ ≈ 0). Accordingly, background distribution *BD*(•) of the region also approaches 0, which means that the region tends to be a salient region.

### Results on datasets

**Fig 14:**
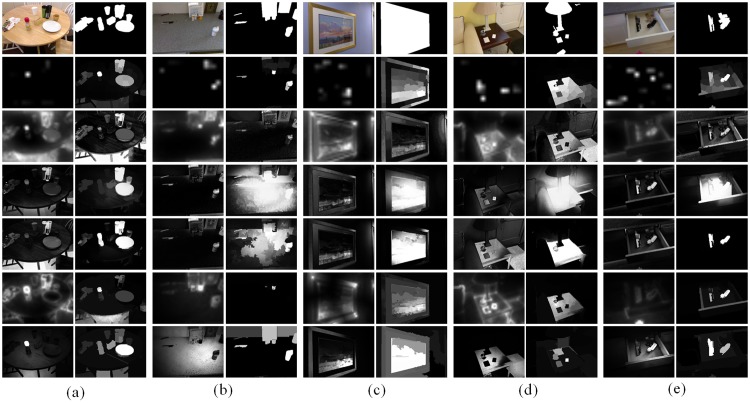
Visual comparison of saliency maps by eleven state-of-the-art algorithms on five different scenes. First column: input RGB image [33], saliency maps by IT [37], GBVS [38], AC [39], FT [25], CA [40], SF [26]; Second column: ground truth [33], RBD [22], RC [20], MB [41], MST [42], GP [43] and ours.

Herein, we first give a visual comparison with eleven different methods (IT [37], GBVS [38], AC [39], FT [25], CA [40], SF [26], RBD [22], RC [20], MB [41], MST [42] and GP [43]) on two dataset(DSD and ECSSD). Fig 14 shows saliency maps and ground truths of five examples on DSD dataset. In the first case, there are several objects on the burlywood table that is placed on the dark floor. We can catch these foreground objects effortlessly and rapidly, but it is a relatively hard task for artificial computational models. For instance, the dark floor belonging to the background area is allocated with the highest saliency value in four models of GBVS, FT, RC, SF and GP, which indicates that the floor is regarded as a salient region. Besides, the middle carton and the right cup against the table of a similar color are difficult to be detected that can be found from all eleven models. Taking advantage of not only color but also depth, spatial layout, boundary connectivity and region roundness, the middle carton is assigned with a medium saliency value in the proposed model. In particular, that the glass bottle with red cap is divided into a whole region profits from the graph-based RGB-D segmentation. MB and MST perform well in the first case, but they sometimes mistake background areas for salient regions, such as the second and forth cases. In summary, our algorithm visually outperforms others in regard to ground truth.

**Fig 15:**
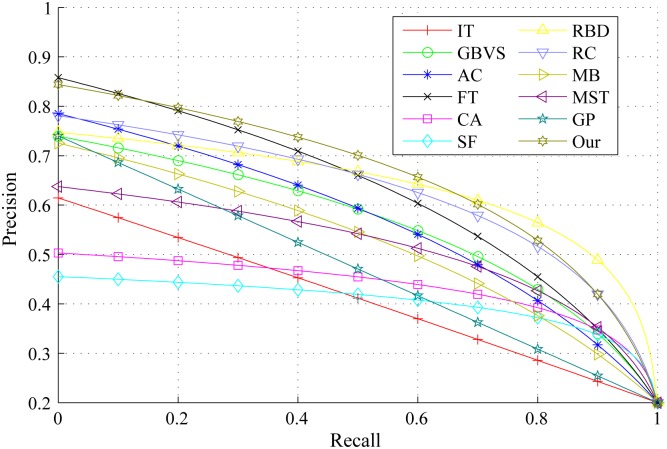
PR curves for different salient detection methods on the DSD dataset.

**Fig 16:**
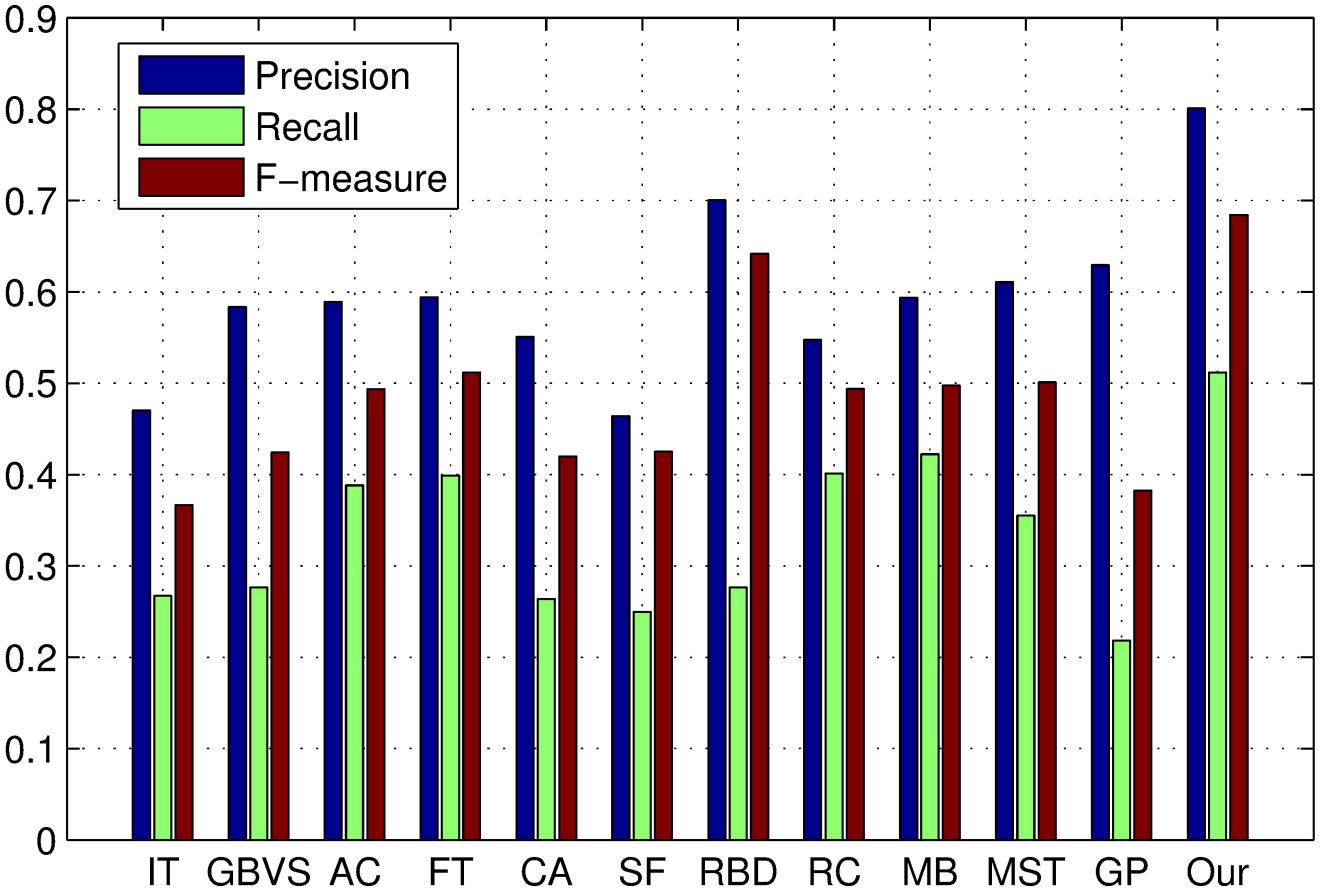
F-measure for different salient detection methods on the DSD dataset. Precision, Recall and F-measure using an adaptive threshold.

**Fig 17:**
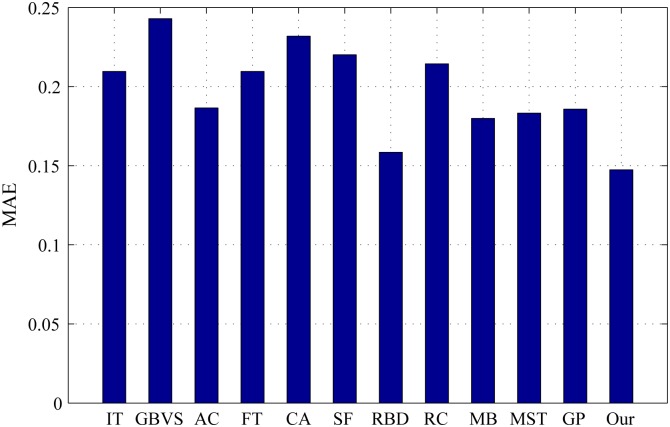
MAE for different salient detection methods on the DSD dataset.

**Fig 18:**
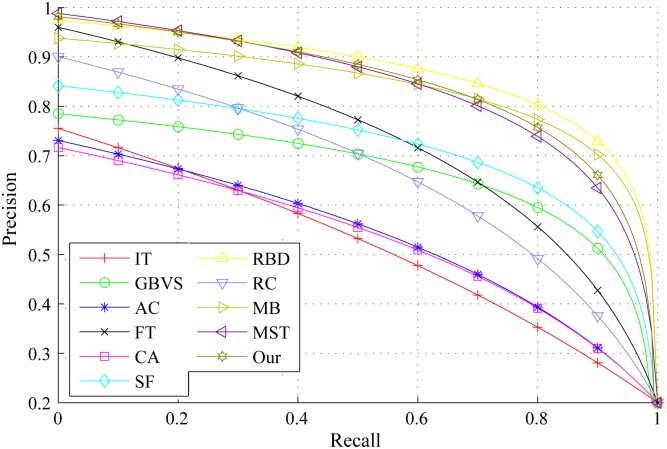
PR curves for different salient detection methods on the ECSSD dataset.

**Fig 19:**
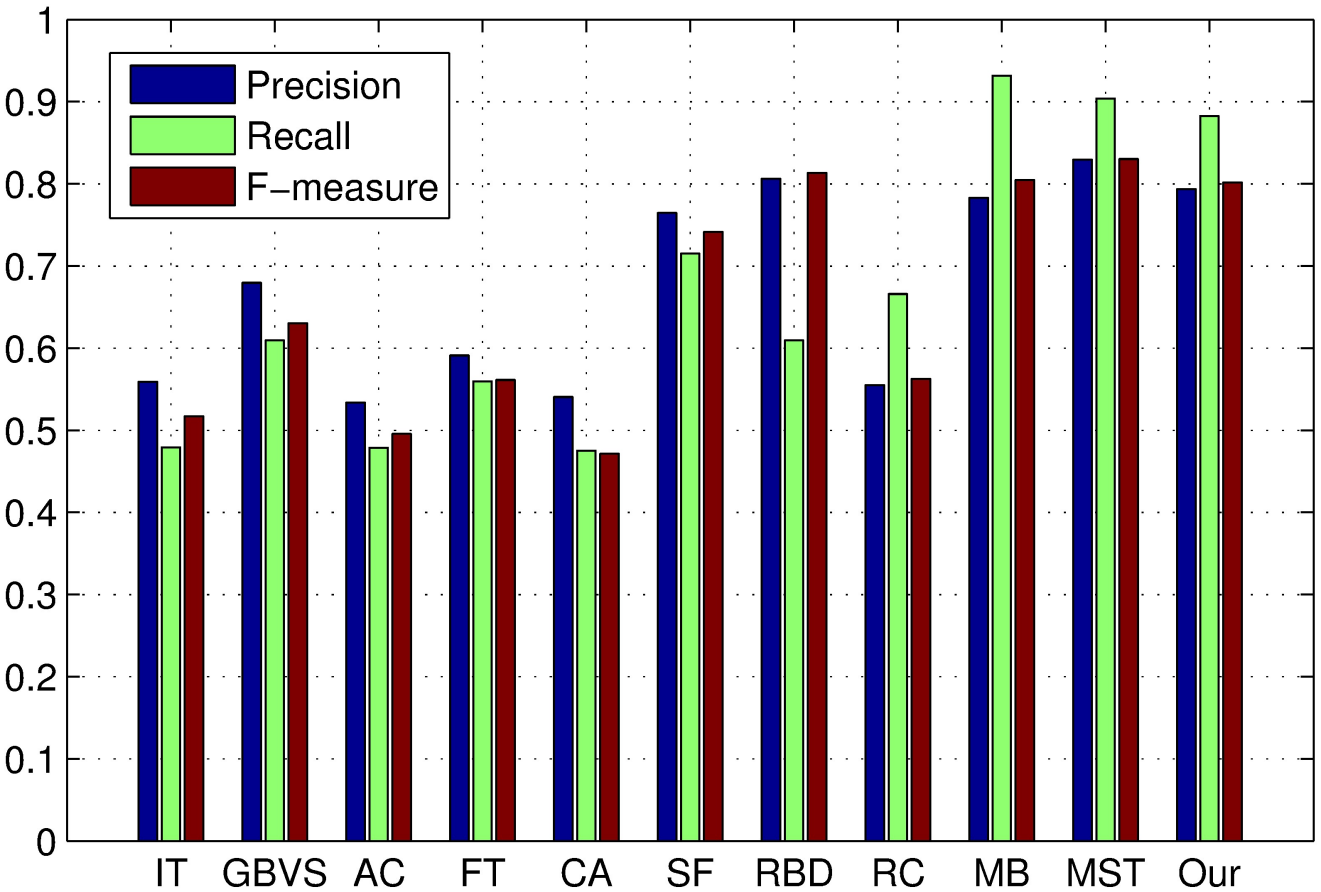
F-measure for different salient detection methods on the ECSSD dataset. Precision, Recall and F-measure using an adaptive threshold.

**Fig 20:**
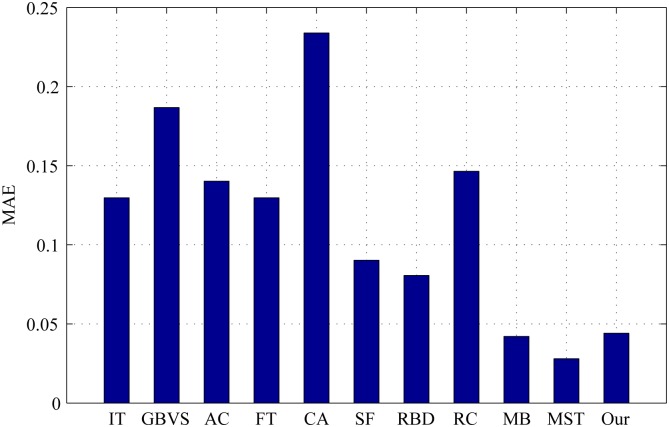
MAE for different salient detection methods on the ECSSD dataset.

The PR curves, F-measure and MAE are obtained to quantitatively evaluate the performance of our method in regard to others. Fig 15 shows PR curves of all the competing approaches on the DSD dataset and it clearly demonstrates that our method performs favorably against other eight. Particularly, the intersecting point of precision 0.2 and recall 1.0, where all pixels are retained as positives, means salient regions occupy 20% of an entire image on average [20]. From Fig 16, it is observed that the proposed approach achieves the best F-measure performance. Consistently, the MAE result of our model has the smallest value on the DSD dataset as shown in Fig 17 which also supports the excellence of our method. Figs 18, 19 and 20 shows PR curves, F-measure and MAE of ten different approaches on the ECSSD dataset, respectively. Note that we don’t consider GP algorithm in the experiments on the ECSSD dataset, because GP algorithm requires depth cues such as depth images and point clouds. From the quantitative comparison with ten models, the proposed model get moderately good performance colosd to MB and MST. As shown in Fig 18, four comparable approaches including RBD, MB, MST and our model outperform the other seven. In Figs 19 and 20, it can be seen that RBD performs poorly compared with MB, MST and our model, and our model underperforms slightly MB and MST. In the ECSSD experiment process, we discover that sometimes our model may not hold the intergrality of foreground objects when they occupy a large area in terms of an image. In summary, our model is suitable for indoor scene with complex background, multi salient objects, while MB and MST algorithm are applicable to natural images with simple background and single salient object.

## Application on the robot platform

### The robot platform

**Fig 21:**
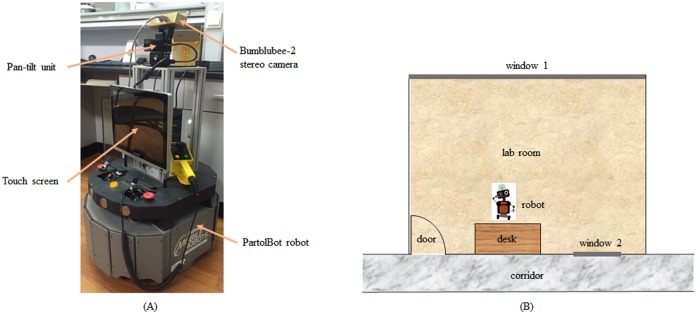
The robot platform and layout of the lab room. A: the robot platform. B: layout of the lab room.

To explore the indoor robot application of our method, a mobile robot platform is built that is equipped with a PartolBot robot, a Bumblubee-2 stereo camera that can capture images at the maximum resolution of 1024*768 pixels, a miniature pan-tilt unit PTU-46-17.5 that can provide accurate real-time positioning of a camera, and an Anmite touch screen that is used as human-machine interface(HMI) providing users with visual inputs and corresponding perception results intuitively. Note that depth cues is only used to assist the pre-segmentation, in spite of the inconsistency of depth generation between the Kinect in the DSD dataset and the stereo camera here. The PatrolBot robot is a programmable autonomous general purpose service robot rover built by MobileRobots Inc, which embeds an onboard computer and also can be connected with an offboard computer by wireless network. For these experiments, we choose the former approach to run the program of our algorithm. A structure made of aluminum alloy is mounted on the robot in order to give it more height, providing the point of view of a child with a height of 1.2m. The software is implemented in C++ using Visual Studio 2008. The robot platform can be seen in Fig 21A.

### Results on the robot platform

For application in an actual environment and performance evaluation of our proposed algorithm, a lot of experiments are conducted on a real robot platform mentioned above.The situation of different viewpoints is challenging for object detection in that appearances of object may differ significantly when the viewpoint changes. Another important property of the visual system is the robustness under illumination variations, and this is a problem that frequently induces difficulties in robotic applications since images often look very different if illumination changes. Moreover, partial occlusions between objects are common cases in daily life which is an essential problem for object detection to be solved. Therefore, we design three different kinds of experimental constructions that are different viewpoints, illumination variations and partial occlusions, referring to [44].

**Fig 22:**
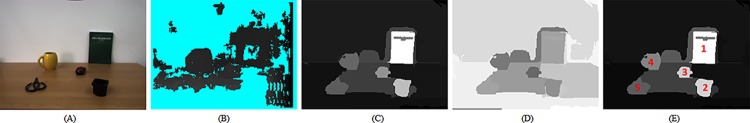
An instance running on the robot platform. A: left camera image captured by the Triclops stereo vision SDK of the Bumblubee-2 stereo camera. B: raw disparity map, computed automatically by the Bumblubee-2, in which the aqua color parts show the areas where the disparities are not available. C: primary saliency map. D: background distribution map. E: final saliency map with manual annotation for the attention shift sequence.

The experiments are conducted in our lab room whose layout is depicted in Fig 21B. The robot is placed in front of a desk on which we arrange several objects such as book, mouse, cup, and so on. These objects’ positions will be changed during the partial occlusion test. There are two windows as shown in Fig 21B: window 1 is a large French window with curtains, while window 2 is a high and small window without curtains along the corridor. So daylight entering from window 2 is rather fewer. Because the room faces the north and lighting is not good, so fluorescent lamps are on throughout the whole phase of robotic application. In consequence, illumination conditions will be controlled by means of opening and closing curtains of window 1. An instance of salient region detection is shown in Fig 22. In this case, the raw disparity map in Fig 22B generated by the Triclops Stereo Vision Software Development Kit(SDK) of Bumblubee-2 is not fully populated, and the missing data is labeled with aqua color. Curtains of window 1 are opening which means the current illumination contains artificial light and sunlight. Since the illumination is not equally distributed over the scene and shadows are present, background areas are segmented into some regions as shown in Fig 22D. Fortunately, five objects are extracted from the image successfully and allocated with high saliency values on account of the excellent primary saliency measure. The attention shift sequence, salient regions in descending order, is 1–2–3–4–5 that is labeled by red number in Fig 22E.

**Fig 23:**
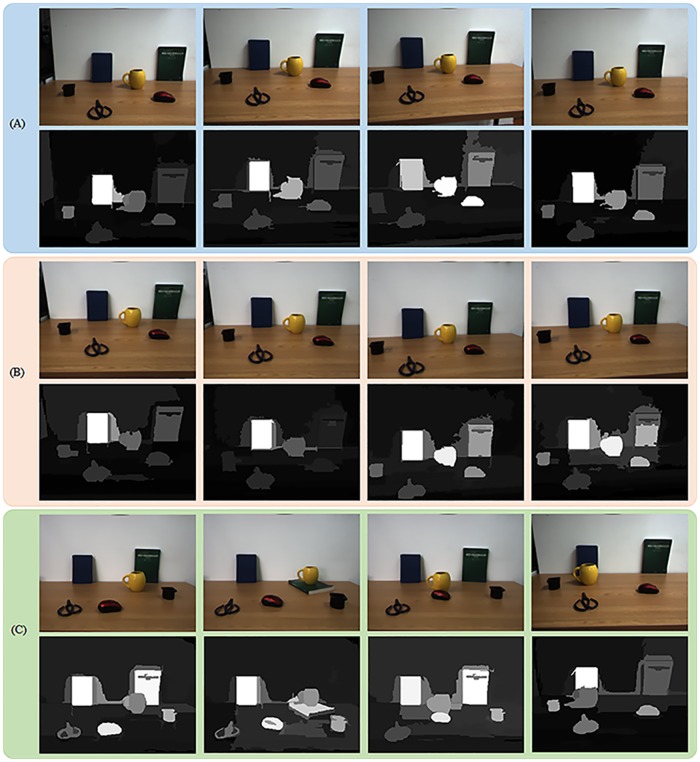
Experiment results of three experimental constructions that are different viewpoints, illumination variations and partial occlusions. The first row of (a): input RGB images from different viewpoints under illumination condition 1. The second row of (a): final saliency maps corresponding to the first row of (a). The first row of (b): input RGB images from different viewpoints under illumination condition 2. The second row of (b): final saliency maps corresponding to the first row of (b). The first row of (c): input RGB images in the first three cases are captured from the same viewpoint under illumination condition 1 while that of the last case is captured from another viewpoint under illumination condition 2. The second row of (c): final saliency maps corresponding to the first row of (c). illumination condition 1: some artificial light and few daylight when curtains of window 1 is closed. illumination condition 2: some artificial light and lots of daylight when curtains of window 1 is open.

The first experiment is a robustness test in regard to different viewpoints, which is implemented by moving wheels and the pan-tilt unit of our robot platform slightly. Herein two separate groups of viewpoint change experiment are displayed in Fig 23(a) and 23(b), whose difference is the illumination conditions that will be discussed in the next experiment. For the Fig 23(a), the left notebook is detected with the first focus in three of four cases. In the third image, the first focus diverts to the yellow cup. This happens probably because centroid of the cup is closer to the image center and its region roundness gives a more weight to background distribution measure. For the Fig 23(b), the left notebook is detected with the first focus in all of four cases. As we can see from these figures, the several objects are detected with high saliency values generally, and the sequence of attention shift oscillates as the viewpoint changes. Therefore, the algorithm has a good detection performance despite slight different viewpoints.

The second experiment is a robustness test in regard to illumination variations, also depicted in Fig 23(a) and 23(b). Two different situations are set up by closing(Fig 23(a)) and opening(Fig 23(a) and 23(b)) curtains of window 1, corresponding to illumination condition 1 and 2 respectively. Note that daylight is unavoidable for window 2 has no curtains, thus illumination condition 1 denotes some artificial light and few daylight while illumination condition 2 denotes the same artificial light and lots of daylight. Segmentation results of illumination condition 2 are inferior to that of illumination condition 1, such as the last instance in Fig 23(a) and 23(b), in that the situation introduces more light interference. Nevertheless, five objects are detected with relatively high saliency values and the first focus attaches to the left notebook generally.

The third experiment is a robustness test in regard to partial occlusion. As shown in Fig 23(a) and 23(c), we give four instances occluding different objects. For the first three instances, the curtains of window 1 are all opening and input images are captured from the same viewpoint. We can see from the first three columns of Fig 23(a) and 23(c) that the saliency of occluded objects is influenced faintly. The forth instance is designed for comprehensive evaluation of the reliability of our model in robotic applications, whose three experimental variables are all different from those three cases. And its first focus is stable on the left notebook although it is occluded by the yellow cup. Overall, the algorithm can allocate high saliency values for dominant objects regardless of occlusion. However, background regions are segmented into splintery ones so that saliency maps are to some extent cluttered in the background regions.

## Conclusions

This paper presents an effective method for indoor robot application to detect salient regions or objects in complex environment. Firstly, to keep the completeness of salient object candidates, a given image is divided into regions by the graph-based RGB-D segmentation using color and depth feature. Secondly, based on the segmentation results, the primary saliency map is measured by utilizing color(distance in RGB color space and color delegation of a region), region area and spatial layout of regions, while the background distribution map is calculated by region roundness, boundary connectivity and spatial layout between image center and region. During this stage, the color delegation of a region is enumerated by building a color histogram and picking more frequently occurring colors. Besides, region roundness is proposed to describe the compactness of a region to produce robust background distribution measure. Thirdly, the final saliency map is calculated by combining the above two maps in an exponential function way, where the regions with the relatively high saliency values are considered as salient in our model. To validate the proposed approach, two kinds of experiments are conducted. The first experiment is carried out by the comparison with eleven significant models on the public DSD and ECSSD dataset, whose results show that our approach outperforms these existing saliency detection approaches in indoor secnes. The second experiment on the self-made robot platform shows that the algorithm is robust to different viewpoints, illumination variations and partial occlusion.

The contributions and advantages of the work are summed up as follows. Firstly, in order to keep the completeness and compactness of salient region candidates, depth cues are utilized during the graph-based RGB-D segmentation stage, which is important for robots to perceive the location and size of desired objects. Besides, assuming that salient regions possess the attributes of compact sizes while background ones tend to distribute widely and near image boundaries, we put forward the concept of region roundness, the representation of how compact a region is. Background distribution measure is more robust when region roundness is applied. Moreover, a principled framework which combines the primary saliency and background distribution is built, and it is applied on the indoor robot platform.

Honstly, there are still some insufficiencies in our model. For example, the graph-based RGB-D segmentation is to some extent susceptible to illumination variations, particularly to shadows, which can be seen from Fig 23. Specifically, segmented salient region candidates are prone to carry rough edges, which weakens values of region roundness, same with the final saliency values. This work is concentrated on the bottom-up, stimulus-driven and involuntary stage of attention, but it is not enough for the robot application. As the further work, we would like to introduce top-down, goal-driven and voluntary stage of attention into the method so that our indoor robot has the ability of “active vision”.
